# Substance over style: is there something wrong with abandoning the white coat?

**DOI:** 10.1136/medethics-2013-101900

**Published:** 2014-07-21

**Authors:** César Palacios-González, David R Lawrence

**Affiliations:** Institute for Science Ethics and Innovation, The University of Manchester, Manchester, UK

**Keywords:** Applied and Professional Ethics, Autonomy, Clinical Ethics, Education, Ethics

## Abstract

In this paper, we address points raised by Stephanie Dancer's article in *The BMJ* in which she claimed that by ‘dressing down’, physicians fail to adhere to the dignitas of the medical profession, and damage its reputation. At the beginning of this paper, we distinguish between two different senses in which a person can be, as she terms it, ‘scruffy’; and then we address Dancer's three main claims. First, we argue that in regard to the medical profession it is fallacious to assume, as she appears to do, that someone is incompetent or irresponsible when such a judgement is grounded in the fact that a physician is not dressed in a formal way. Second, we argue, contrary to her claim, that the dignified nature of the medical profession is in no coherent way linked to sartorial elegance or lack thereof, but rather, that such dignity is bound to the value of the medical practice in itself, to patients, and to society at large. Third, we examine two ways in which doctors can ‘dress down’ and show that ‘scruffiness’ does not necessarily intimates a lack of personal hygiene. Finally, we show that pointing to mere statistical correlation without causation, cannot be used as an argument against scruffiness. We conclude by suggesting that in the medical context, it is more appropriate to educate patients than to chastise practitioners for not following arbitrary cultural mores.

## Introduction

Stephanie Dancer, a UK consultant microbiologist, recently called for doctors to stop dressing down and to dress up. In a ‘personal view’ article published in *The BMJ*, she argued that ‘scruffy’ doctors damage the medical profession's reputation, and that doctors’ scruffiness is an indicator of a decline in their hygiene practices.^1^

Dancer's point of view appears to have originated (or at least reached a tipping point) in what she considers the *non-conclusive science* and supposed *political motivation* that backed up the mandatory dress code guideline published by the UK Department of Health (DOH) in 2007 (and which was revised in 2010 in order to include further advice on cultural issues related to workwear).^1–4^ This dress code guidance demands that physicians roll up their sleeves, and remove their ties, watches and white coats.

Although doctors’ apparel is not a central debate topic in bioethics, Dancer's piece is worthy of reply largely because it incarnates the common belief, and prejudice, that there is something morally wrong when young professionals decide not to follow the social traditions associated with the dignitas of certain professions, in this case by not wearing a particular type of clothing. Traditionally, or at least for the last 80 years, the attire that is regarded as appropriate for western physicians is formal wear plus white coat (henceforth: formal wear): for women it is tailored trousers or skirt, shirt, and white coat; and for men dress trousers, shirt, neck tie, and white coat.

Dancer makes her case in favour of dressing up and keeping the white coat and formalised dress by advancing two arguments against ‘scruffiness’. The first is that medicine is a distinguished profession and physicians should dress accordingly with its status^1^; implying that ‘scruffy’ doctors do not embody the dignitas associated with the profession. Moreover, according to her, dressing down diminishes physicians’ image as responsible and competent. The second argument that Dancer advances is that scruffiness is a sign of poor personal hygiene and that this behaviour imperils patients by means of less vigorous infection control. She concludes her article asserting that scruffy doctors should change their habits and start (1) dressing formally and in accordance with the dignity of the medical profession and (2) should improve their hygiene.

Before dissecting Dancer's claims, it is important to bear in mind that the term ‘scruffiness’ has at least two broad meanings in vernacular usage: the first refers to the condition of being ‘unclean’ and the second refers to ‘not being arranged stylishly’. In the following sections, we examine how both meanings relate to physicians’ attire, and if there is a distinct moral obligation for physicians to dress in a specific way.

## Scruffiness as clinicians wearing informal attire

In this section, we examine stylishness, and whether, in fact, doctors’ ‘scruffy’ clothes (informal attire) do affect their image as responsible and competent. We also assess the connection between the dignitas of the medical profession and formal attire.

When Dancer states that physicians presenting themselves ‘poorly’ erode their image as responsible and competent, she is making an empirical claim.^1^ The problem with this claim is that she does not provide robust empirical support for it (even though, because she is advancing the claim, she has the burden of proof). In point of fact, Dancer omits to mention that there are several empirical studies on how patients and physicians perceive physicians’ competence and degree of responsibility in relation to how they dress. She also failed to mention that the results of the bulk of research that has been carried out on such matters in no way suggest a unified viewpoint.

In order to present a more complete view, what should be said regarding the empirical evidence is that while on the one hand some of the results of the vast amount of scientific literature available on the subject show that some patients perceive doctors as more competent and responsible when they are dressed formally and with a white coat^5–11^, other results on the subject conclude that some patients perceive doctors dressed informally as equally so.^12–18^ This has not gone unremarked; at least three other papers acknowledge this contradictory multiplicity of empirical results and they tend to conclude that physicians should choose their attire prudentially.^19–21^

Given that there is a wealth of data and all of it is easily available we could suggest that, at best, Dancer's point is either skewed due to its reliance on only one research paper and a newspaper article that fails to present any new findings (and which it must be noted has a known conservative bias),^1^ ^22^ or she is cherry-picking results in order to defend her position regarding formal attire. It is also worth acknowledging that even the authors of the academic article that Dancer cites recognise that one of the limitations of their study was: ‘that it is a single-center study conducted at one US Veterans Affairs medical center, and veterans and visitors may have chosen the professional attire due to their previous comfort level with the military uniform.’^5^ It is perhaps significant that even the authors realise—and imply—that their conclusions should not be extrapolated to all patients’ perception of physicians’ attire.

In short, then, the significant point for this debate is that there is no unified take on the matter. This means at least two things: first, that anyone who states that *all* patients (or ‘patients’ as a collective) perceive informally dressed doctors as less competent and responsible has a burden of empirical proof which it is not possible to deliver. Second, given that some patients regard ‘scruffy’ doctors as competent and responsible, it is also not true that *all* patients perceive that doctors’ dress can erode their image as responsible and competent.^1^

At this point, it is valuable to consider whether doctors’ attire is in any way related to the dignity of the medical profession. We might ask, however, for some clarification of just what this idea of dignity refers to. We could advance the proposition that ‘dignity’ here refers to *the value of the medical practice in itself, to its patients and to society at large*. The underlying question, then, is: if the everyday attire of physicians changes, for perceived good or bad, what does this mean for the value of their profession?

The answer is that there is no attire that a physician could wear that could change how important the medical practice is to patients or to society. The adequate treatment of a fracture has inherently the same value regardless of the sartorial choices of the provider of the treatment. The fact that nineteenth-century doctors wore tailcoats and formal headwear, and that twenty-first-century doctors might wear scrubs is irrelevant to the value of the medical practice. Therefore, we can safely assume that doctors’ attire has no bearing on the inherent dignitas of the medical profession, even where the doctor's appearance is, for whatever reason disagreeable to the patient, this does not change the value of the medical intervention to the health of that patient; and we have already established that this value is the root of medicine's cachet. To state otherwise would be the same as to state that the value of a doctor's medical practice fluctuates with each patients’ perception of the physicians’ attire.

It might be possible to agree that doctors’ attire is unrelated to the value of the medical profession and at the same time advance that clinicians’ attire is morally relevant because it can provide the patients with psychological ease, by means of satisfying the patients’ idea of how a competent and responsible physician should look. However, such a claim rests on an epistemic error. The mistake lies in that one cannot ascertain others’ professional skills and character simply by examining their clothing.

A rejoinder to this could be to suggest that it is impossible to imagine a paragon of ‘doctorly virtue’ in ‘scruffy’ outfits because a competent and responsible doctor, by necessity, dresses formally. With such an idea we could question whether the respondent could imagine a competent and responsible physician on the battlefield, dressed in full armoured gear, while properly medically attending to a fellow soldier. If they answer that actually they can, then we have demonstrated that it is indeed possible to imagine a doctor with the desirable characteristics and attitude dressed in non-formal (in the white coat sense) attire, and that in no way is there a necessary relationship between being competent and responsible and dressing formally.

With the mention of military uniform, it is perhaps worthwhile to very briefly address the importance of prescribed dress to the wearers themselves; for instance, a soldier may comport him/herself very differently when in uniform and when in civilian clothing. The same could be suggested to apply to our doctors, in support of their wearing the white coat. However, those who would claim this latter view have the burden of proof to either present empirical data that shows that dressing in a certain fashion alters the wearer's competence and degree of responsibility to a significant extent (and for which our research turned up no evidence).

Returning to the main point, someone could consider that the explanation regarding the epistemic error is accurate; but at the same time they could claim that the role of a physician is not to redress patients’ epistemic errors, but to treat patients in accordance with the medical sciences while providing as much comfort as possible. If this was true, so they might claim, then physicians are required to dress in a way that puts their patients at psychological ease while at the same time providing them with appropriate medical treatment. To this we answer that patients’ potential psychological unease about a doctors’ informal attire is *not a strong enough reason* to trump physicians’ autonomy in relation to how she or he might dress (if indeed this potential is even sufficiently extant—as discussed previously—and this is far from being evident). In this sense, and this is our main point, *the limits on physicians’ attire need only be when they medically endanger patient*s (eg, when a physicians’ dress acts as a vector for pathogenic transmission, an idea we will explore shortly) *and the societal contractual limits on harm and freedom of expression*. This means that doctors do not have an obligation to dress formally when dealing with their patients. In this respect, we abide by the liberal tradition in that interference with individual liberty is permissible only to prevent harm to others.

For example, we do not think that a patient's feeling against female doctors wearing something else than long skirts should be accompanied by forcing female doctors to only wear skirts during working time. Doctors should not be obliged to dress *as society, or the majority, requires* if there are not good and strong moral reasons for doing so (we are not excluding that doctors might freely enter into certain work contractual obligations regarding mandated dress code). Second, we think that forcing doctors to dress formally in order to comply with ‘socially acceptable’ ways maintain and supports harmful stereotypes (eg, for the above instance, gender and class stereotypes about how professional women should look and dress) that have no place in liberal societies. It is true that educating patients that doctors’ attire is irrelevant to their medical practice might take time and effort, but this is no different from when patients were taught that female or non-white doctors were as fit for medical practice as white male doctors (with these examples, we are not of course saying that such cases are morally on par with allowing doctors to dress as they please, merely that all social changes take effort, and respecting doctors’ autonomy and fighting harmful stereotypes is worth this effort). This is not to say that doctors may not find and subscribe to other reasons (be they practical or conventional) to refrain from dressing in ways that others might find insulting, disrespectful, indecent, or hostile. What should be clear, however, is that this decision should rest on a personal compromise that doctors autonomously embrace. It is important to remark here that dressing formally (or in a way that pleases the patient) is a supererogatory action, in the sense that it is an action of consideration towards the patient which could be regarded as good but it is not stringently required.

## Scruffiness as poor hygiene

In this section, we examine the relationship between scruffiness and poor hygiene habits, whether ‘scruffiness’ (as in informal wear) necessarily intimates a lack of personal hygiene.

After the DOH issued a guidance regarding uniforms and workwear, now popularly known as the ‘bare below the elbows’ guidance, there was discussion in academia regarding the scientific validity of what appeared to be its main claim: that physicians’ attire and workwear play a role in spreading infection and is related to the great number of hospital-acquired infections (HAI) in the UK medical centres.^2^ ^23–28^ This discussion was product of the DOH's assertion ‘[a]lthough there is no conclusive evidence that uniforms and workwear play a direct role in spreading infection, the clothes that staff wear should facilitate good practice and minimise any risk to patients.’^4^ Due, we believe, to this assertion, Dancer claims that the bare below the elbows guidance appears to have been enacted more as a political gesture than a real attempt to tackle the problems of UK hospitals and HAIs.^2^ According to her, although such measures are trying to impose good medical practice, they actually are short-term solutions that do not adequately address the longer-term problems with HAIs.^2^

So, if there is non-conclusive evidence that physicians’ attire plays a role in spreading infection, then how does scruffiness relate to HAIs? According to Dancer, the link between the two is that scruffiness is a sign of poor personal hygiene, she claims: ‘Scruffiness, however defined, also intimates a lack of personal hygiene and corresponding lower standards of hygienic behaviour.’^1^ Now, if Dancer is not claiming that there is a necessary relationship between the hygienic habits of doctors and how they dress, then she might be claiming that there is such between informal clothing and the transmission of pathogens. This would mean that there is something inherent in the physicality of informal clothing that causes it to be more unhygienic than formal clothing. If this is what Dancer is claiming, then it is important to realise that wearing informal clothing can be understood in two different ways. In the first, wearing informal attire should be considered as wearing formal attire minus white coat, tie, watch and accessories (dressing down in a more literal sense). It could also be understood as doctors wearing a style and type of fabric that is expressly not formal, and that has never been used while attending patients. This distinction is important because each provides different conclusions. If we are talking about the second interpretation, then we should accept that this line of argument is open to empirical verification (eg, whether denim is a better fomite than wool), and that the acceptance of wearing a certain textile would be conditionally related to whether it more easily facilitates the transmission of pathogens or not. If there was an instance of this case, this would not mean that there is a case against scruffiness (in the first sense) in general; but it would mean that doctors should refrain from using certain type of fabrics in regard for their patients’ safety. Conversely, if she is talking about the first interpretation of informal dress (ie, literal dressing down from formal dress) then she could not claim that this is causally related to the transmission of pathogens. Why? Because, as she says ‘Is there any evidence that staff apparel has been implicated in the transmission of pathogens to patients? None at present, although all clothes, including ties, may be covered with a range of microbial flora.’^1^ If, in fact, there is no evidence—as she admits—that clinicians’ normal apparel (formal attire) is implicated in the transmission of pathogens to patients (while acknowledging that apparel can *transport* them and that there is evidence that ties, white coat, identity tags, and clothes in general are covered with pathogens, and that removing the white coat improves wrist washing^25^ ^26^ ^29–31^), then it is irrelevant whether doctors dress down (in the literal sense) or retain their traditional workwear. If it turned out to be that clothing materials and configuration are irrelevant to the transmission of pathogens, and what is instead important is that they are washed, clean and that they do not come into contact with the patient unless this is necessary for the medical treatment, then the fashion tendencies that doctors decide to follow should be considered as morally irrelevant (taking into account the fashion limits that we mentioned earlier) insofar as they fulfil these criteria.

At this point, we should query to what extent doctors should go in order to try to avoid the risk of harm to their patients from their clothing. We think, along with the DOH, that even when there is no conclusive evidence that physicians’ attire plays a direct role in spreading infection, the fact that dressing down removes possible fomites makes it a sensible and commendable act given that it minimises risks for the patients. The loss to physicians who wish to wear formal attire is outweighed by the patients’ gain from being in contact with fewer possible sources of infection. This account for dressing down—reducing the possible number of fomites—differs from the case of dressing up for the psychological ease of some patients in that the patients’ loss of ease is, as discussed previously, far from a direct or serious medical harm. Following the same line of argument, and given that patient care is paramount in medical practice, if antibacterial clothing (or some other infection-controlling fabric) were ever proven effective, then doctors would actually be morally obliged to wear such materials, just as they are morally required not to wear contaminated scrubs in surgery.^32^

A particular concern is one of practicality. It could be thought that informal dress would make it more difficult for patients to identify clinicians. Here, Dancer's claim makes sense: ‘I hear patients complain that they do not know who the doctor is: no tie, no white coat, no jacket, and no presence.’^1^ Except for the ‘presence’ part, which we hope we have refuted, we agree with the patients’ sentiment. If doctors are going to move from formal attire to other types of clothing, then it should be made a priority that patients are capable of identifying them. Suggesting means is perhaps fodder for a different discussion, though a clean stethoscope, along with clean standard visible identity badges could go some way to solving this issue.

## A question of statistics

During review, the possibility was suggested to us that both of Dancer's claims could conceivably be justified from a statistical point of view. This means that even if there is no necessary casual relation between being a ‘scruffy’ doctor and being irresponsible, incompetent and unhygienic, it could be that *statistically*, scruffy doctors are more irresponsible, incompetent and unhygienic than non-scruffy doctors. The reviewer noted that if this was the case, then patients’ unease about being attended to by a ‘scruffy’ doctor would seem justified. Now, does this appeal to statistics undermine our defence of ‘scruffy’ doctors? We do not think so; indeed, there are good reasons to doubt this worry.

First of all because those making such a claim have the burden of proof to show that such statistical correlation exists and that it is statistically significant (and, as far we know, there is no such empirical data). Second, even if the statistical data did exist and was significant, the fact that there is only correlation without causation (as we have discussed earlier) would still mean that it would be an epistemic mistake to prescribe that doctors should wear formal attire on the basis that this would improve doctors’ responsibility of action, respectability and hygiene habits. Actually, what this statistically significant data would demand is a search for the unknown cause that makes doctors irresponsible, incompetent and unhygienic, and deal with it in such way that the behaviours are eradicated. Doing otherwise (demanding that doctors dress in a certain fashion because of this statistical data) would be to guide our action on the basis of the cum hoc ergo propter hoc fallacy, and this would not solve anything!

## Conclusion

Here, we have examined Dancer's central concerns over trends in doctors’ dress, namely, that what she sees as a decline in sartorial standards is damaging to the august nature of the medical profession and potentially damaging to patients’ health. We found that her arguments are flawed because there is no empirical evidence that support them, but more importantly because they unjustly burden physicians to adjust their dressing to accord with arbitrary societal mores. In secular pluralistic societies, instead of trying to coerce doctors into dressing formally for the sake of tradition, we should actually educate patients about the importance of infection control and how this relates to doctors’ attire (just as they were hopefully taught that the clinicians’ sex, race, or sexual orientation is not important for their medical care). We should also educate patients that what is important for their medical treatment is not a white coat, or a tie around the doctor’s neck, but the doctor’s competence and good practice. Finally, it should be said that if certain specific modes of dress are, after all, related to the dignity of certain professions, then these authors at least would not object *too* strongly to an insistence that philosophers dress in togas just as did Plato, Aspasia, Hypatia, or Aristotle. We wonder if critics of our position would feel the same.
